# Trends in immunological markers of transfusion transmissible infections among blood donors in Mamfe District Hospital, Southwest Cameroon

**DOI:** 10.1186/s12879-024-09119-0

**Published:** 2024-04-02

**Authors:** Sen Claudine Henriette Ngomtcho, Olive Njike Ngo Biyong, Timothy Amos Ekwere, Jonas Merlin Wandji Takemegni, Henrietta Mbah, Sandra Maella Makamdoum Bogne, Omer Aurelle Nkengkanna, Henri Lucien Fouamno Kamga

**Affiliations:** 1 Department of Microbiology, Immunology and Hematology, Faculty of Medicine and Pharmaceutical Sciences, University of Dschang, Dschang, Cameroon; 2 Molecular biology and serology units, National Public Health Laboratory, Ministry of Public Health , Yaoundé, Cameroon; 3 Mamfe District Hospital, Mamfe, Cameroon; 4 Department of Haematology, University of Uyo/University of Uyo Teaching Hospital, Uyo, Akwa Ibom State Nigeria; 5 Department of Biomedical Sciences, Faculty of Health Sciences, University of Buea, Buea, Cameroon; 6 Department of Medical Laboratories Sciences, New-bell District Hospital, Douala, Cameroon

**Keywords:** Blood transfusion, Blood donor screening, TTIs, ELISA, Mamfe District Hospital

## Abstract

**Background:**

Blood transfusion is associated with exposure to blood Transfusion Transmissible Infection (TTIs). The threat posed by the blood-borne pathogens is disproportionately distributed in different healthcare facilities in Cameroon. Thus, there is a need for continuous surveillance of TTIs in the country. This study aimed to assess the screening procedure for blood transfusion and determine the trend in immunological markers of TTIs among blood donors at the Mamfe District Hospital.

**Methods:**

A prospective descriptive, cross-sectional and analytical study was conducted at Mamfe District Hospital from March to May 2022. A total of 165 blood donors were recruited by the consecutive sampling method. Donors were screened using both Rapid diagnostic tests,*T. pallidum* haemagglutination test and indirect enzyme-linked immunosorbent assay (ELISA) for the detection of TTIs. Data generated was entered into an Excel spreadsheet and analysed using the statistical software R, version 4.2.0. Statistical analysis included descriptive statistics of percentages, means ± standard deviation, and student t-test was used to compare both diagnostic techniques, and was considered significant when *p* < 0.05.

**Results:**

A hundred and sixty-five donors were enrolled in the study with a male preponderance giving a male-female sex ratio of 22.5 and a mean age of 32.23 ± 8.60 years. The majority (75.2%) of the donors were of the O-positive blood type, repeat donors (69.1%) and were mainly family replacement and paid donors as against the voluntary blood donors (39.4% and 37.0% vs. 23.6% respectively). overall TTIs prevalence was 18.78% (31/165) (), with HBsAg being the most predominant marker at 12.12% (20/165) followed by *Treponema pallidum*, HCV and HIV antibodies at 4.85 (8/165), 1.21%(2/165), 0.60% (1/165) respectively. Except for the HBV, The prevalence of TTIs was higher when using a single RDT than the ELISA test, and the difference was significant (*p* < 0.05).

**Conclusion:**

Bloodborne pathogens remain a major menace to safe blood transfusion practice in Mamfe district hospital and their detection could be easily missed if the RDT method alone is used for donor screening. Therefore, the donor screening protocol in Mamfe District Hospital should systematically incorporate a confirmation diagnostic test such as ELISA.

## Background

Blood transfusion is an integral and life-saving procedure of modern medical science since the discovery of human whole blood transfusion in 1818 by Dr. James Blundell [1]. It remains an important therapeutic option in many of the life-threatening diseases and also in sustaining life after severe blood loss.

In high-income countries like the United States and many European countries, due to continuous implementation and improvement of more sensitive serologic methods and nucleic acid amplification test (NAT), the residual risk of TTI transmission decreased in 2000 to less than 1: 250,000 for hepatitis C virus (HCV) and 1: 1.3 M for HIV [1, 3]. This however contrasts the findings in many low and middle-income countries (LMICs) where TTIs appear to be more prevalent [2, 3, 4].

Unreliable supply of test kits, and lack of capacity building of laboratory personnel and infrastructures can be some of the factors which could have led to this issue [5, 6].

Furthermore, the majority of blood donors in SSA are either family replacement donors or paid donors [7]. Several documented evidence have shown that blood from these donors are relatively unsafe and less free from TTIs when compared with those from voluntary non-remunerated donors (VNRD) [8, 9]. The VNRD are the recommended blood donors by World Health Organization (WHO) and they form a significant proportion of blood donors in developed countries [7].

In Cameroon, like many other countries in SSA, blood transfusion services are still largely hospital-based, thus the prevalence of TTIs varies depending on the geographical location and sophistication of transfusion practices in that hospital or district. A study in an urban hospital in Douala reported that 13.7% of the tested donors were positive for at least one of the TTIs [Human immunodeficiency virus (HIV), Hepatitis B virus (HBV), Hepatitis C virus (HCV) and Syphilis]. Amongst the infected participants, 8.3% were voluntary donors while 14.3% were family replacement donors [10]. A similar study at the Bamenda hospital-based blood services reported a seroprevalence rate of 10.5% for the four conventional TTIs markers [11]. Studies from other SSA countries with similar demographics have reported similar findings in Kenya, Nigeria and Ethiopia [12–15].

Transfusion of unsafe blood poses a health challenge not only to the recipients but also to the larger society as it adds to the cost of healthcare. Therefore, continuous evaluation of the burden of TTIs among blood donors will help generate evidence-based data upon which protocol for enhancing donor selection strategies and blood safety surveillance systems can be established. Therefore, the objectives of this study were to unveil the demographic profile of blood donors and; determine the trends in immunological markers of TTIs at the Mamfe district hospitalin the South West region of Cameroon.

## Methods

### Study site

This study was carried out at Mamfe District Hospital (MDH), which has an hospital-based blood bank. MDH is a public institution situated at latitude: 5°45’4.12’’N and longitude: 9°18’5.5’’E in the Mamfe subdivision, Manyu division, South West region of Cameroon. It serves as a referral hospital to 14 functional satellite health centres within the Manyu division, located along the Besong Abang-Ekok highway, the trans-African highway truck 6 bordering the Federal Republic of Nigeria.

### Study design

This was a prospective cross-sectional, descriptive and analytical study designed to achieve the set objectives of the study.

### Study period

Blood samples of all consented prospective blood donors recruited at the MDH between March to May 2022 were used for this study.

### Study population

These were prospective blood donors recruited at the hospital-based blood bank of MDH between March and May 2022.

### Study criteria

#### Inclusion criteria

Prospective blood donors had to meet the following conditions: between 18 and 60 year of age, weighing at least 50 kg, presenting at the MDH for blood donation; More over, the medical history of these participants had to fit the requirements of WHO guidelines on assessing donor suitability for donation [16] and National Blood Transfusion Service protocol [17]. All participants had to provide written consent before recruitment.

#### Exclusion criteria

Potential donors were excluded if they donated blood on an interval less than 3 months, were anaemic, had a history of jaundice, malaria, asthma, high-risk (have had unsafe intercourse, drug abuse, tattooing), had history of HBV, HCV, HIV infection.

#### Sampling method and donor recruitment

The participants were consecutively recruited into the study within the study period (March–May 2022). Before the participants were recruited for the study, a community sensitization campaign was carried out within the Mamfe region and its environs on the therapeutic importance of blood transfusion and the benefits of blood donation to the donors via the local radio station in the subdivision. Also, health talks on healthy living for blood donors were organized at the MDH waiting hall for all prospective donors. All participants who met the inclusion criteria were enrolled in the study.

#### Screening procedure

**Fig. 1:**
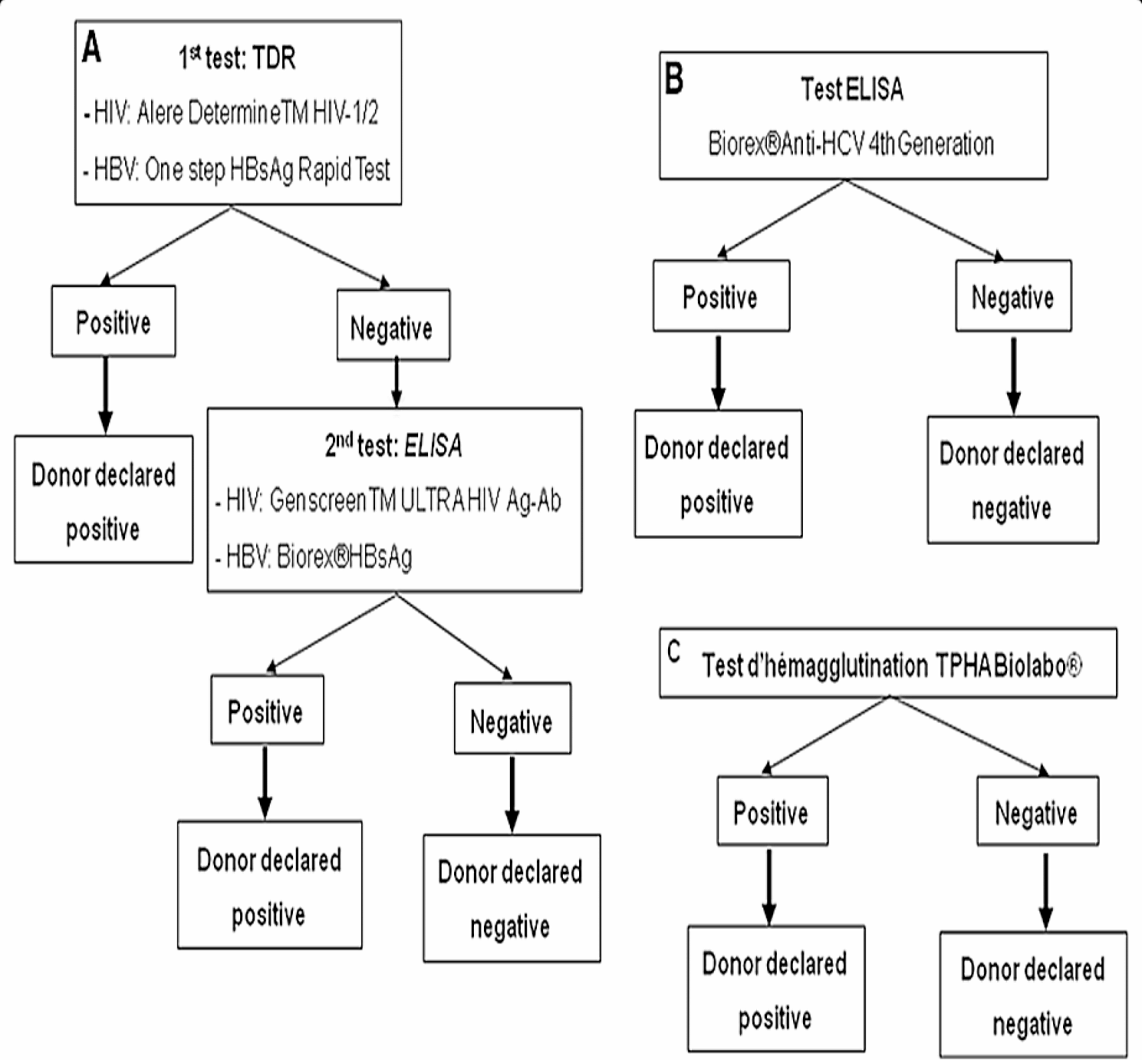
National blood transfusion service (NBTS)-TTI recommended screening algorithm [17]

The National blood transfusion service (NBTS)-TTI recommended screening algorithm (Fig. 1) was used for the following assays: Hepatitis-B surface antigen (HBsAg), Enzyme Linked Immunosorbent Assay (ELISA) anti-Hepatitis-C virus antibodies (anti HCV) and HIV antibody-ELISA 4.0 fourth generation, *Treponema Pallidum* Hemagglutination Assay (TPHA) and Malaria parasite rapid diagnostic test (RDT).

About 5 mls of whole blood was collected by venipuncture from all consenting eligible blood donors into plain dry tubes (Sarstedt, Nümbrecht, Germany) under aseptic conditions. The tubes were centrifuged at 3500 rpm for 5 min after which serum was separated, then transferred into an Eppendorf tube labelled with a unique identification number assigned to that participant and stored in a refrigerator at − 20^0^C. Each sample was stored in duplicate. The stored serum was tested across the RTD and ELISA simultaneously for the detection of HBsAg, HIV antibody, anti HCV and *Treponama pallidum pallidum* antibody (Biolabo®).

For HIV, we used the Alere Determine™ HIV-1/2. For HBsAg and HCV antibodies we used Fortress HBs Ag for the direct ELISA test and Fortress HCV for the indirect ELISA test. All the procedure was done and cut off values determined following the instructions of the manufacturers. The overall prevalence of TTIs was calculated as the number of TTIs positive donors to the total number of donors in the study.

### Data analysis

Data was collected, entered into an Excel spreadsheet and analysed using the statistical software R version 4.2.0 [18]. Statistical analysis included descriptive statistics of percentages, means ± standard deviation; t-test and chi-square difference was considered significant at *p* < 0.05.

### Ethical consideration

All participants were fully informed about the study and only those who provided written consent were recruited. The protocol was approved by the Cameroon National Ethical Committee for research on Human Health before the commencement of the study.

## Results

### Description of socio-demographic characteristics of the study population

A total of 165 eligible blood donors were enrolled in this study: 95% males while a little less than 5% were females. The predominant age group among the donors were those between the age brackets of 18–33 years (56.4%). Many (25.5%) of the donors were farmers, while the least represented occupations were accountants and teachers (4%, and 5%) respectively. In addition, more than half of the donors (54.6%) were unmarried (Table 1).

**Table 1:** Socio-demographic characteristics of the study population

Variables	Sub-variable	Frequency (N)	Percentage (% _95_CI)
Gender	Female	7	4.24 (2.02–8.69)
	Male	158	95.8 (91.3–98.0)
Age group	(18–33)	93	56.4 (48.6–63.8)
	(34–49)	69	41.8 (34.5–49.6)
	(50–60)	3	1.82 (0.581–5.55)
Occupation	Accountant	4	2.42 (0.903–6.34)
	Businessman	19	11.5 (7.43–17.4)
	Driver	39	23.6 (17.7–30.8)
	Farmer	42	25.5 (19.3–32.7)
	Medical Professional	16	9.70 (6.00–15.3)
	Student	21	12.7 (8.41–18.8)
	Teacher	5	3.03 (1.26–7.13)
	Technician	19	11.5 (7.43–17.4)
Marital status	Married	72	43.6 (36.2–51.4)
	Single	93	56.4 (48.6–63.8)

### ABO Blood group distribution and donor characteristics

A significant proportion (75.2%) of the blood donors were of the Rh O + blood type. The remaining (25%) comprise the other blood types in descending order of occurrence (A+-15% >B+- 13%). None of the donors were of the AB blood type. Also, the majority (39.4%) of the donors were family replacement donors while 37.0% were paid donors. Voluntary non-remunerated donors were the least represented (23.6%). In addition, the majority of the donors were first-time donors (30.9%) (Table 2).

**Table 2:** ABO blood group distribution and donor characteristics

	sub-variable	Frequency (N)	Percentage (% _95_CI)
Blood group	A+	15	9.09 (5.53–14.6)
	B+	13	7.88 (4.60–13.2)
	O-	13	7.88 (4.60–13.2)
	O+	124	75.2 (67.9–81.2)
Type of donor	Family	65	39.4 (32.2–47.1)
	Paid	61	37.0 (29.9–44.7)
	Voluntary	39	23.6 (17.7–30.8)
Number of previous donations in the last one year	First time	51	30.9 (24.3–38.4)
	once	23	13.9 (9.40–20.2)
	Twice	16	9.70 (6.00–15.3)
	≥ 3	32	19.4 (14.0–26.2)
	Life time	43	26.1 (19.9–33.4)

### Overall seroprevalence of TTIs using ELISA

**Fig. 2:**
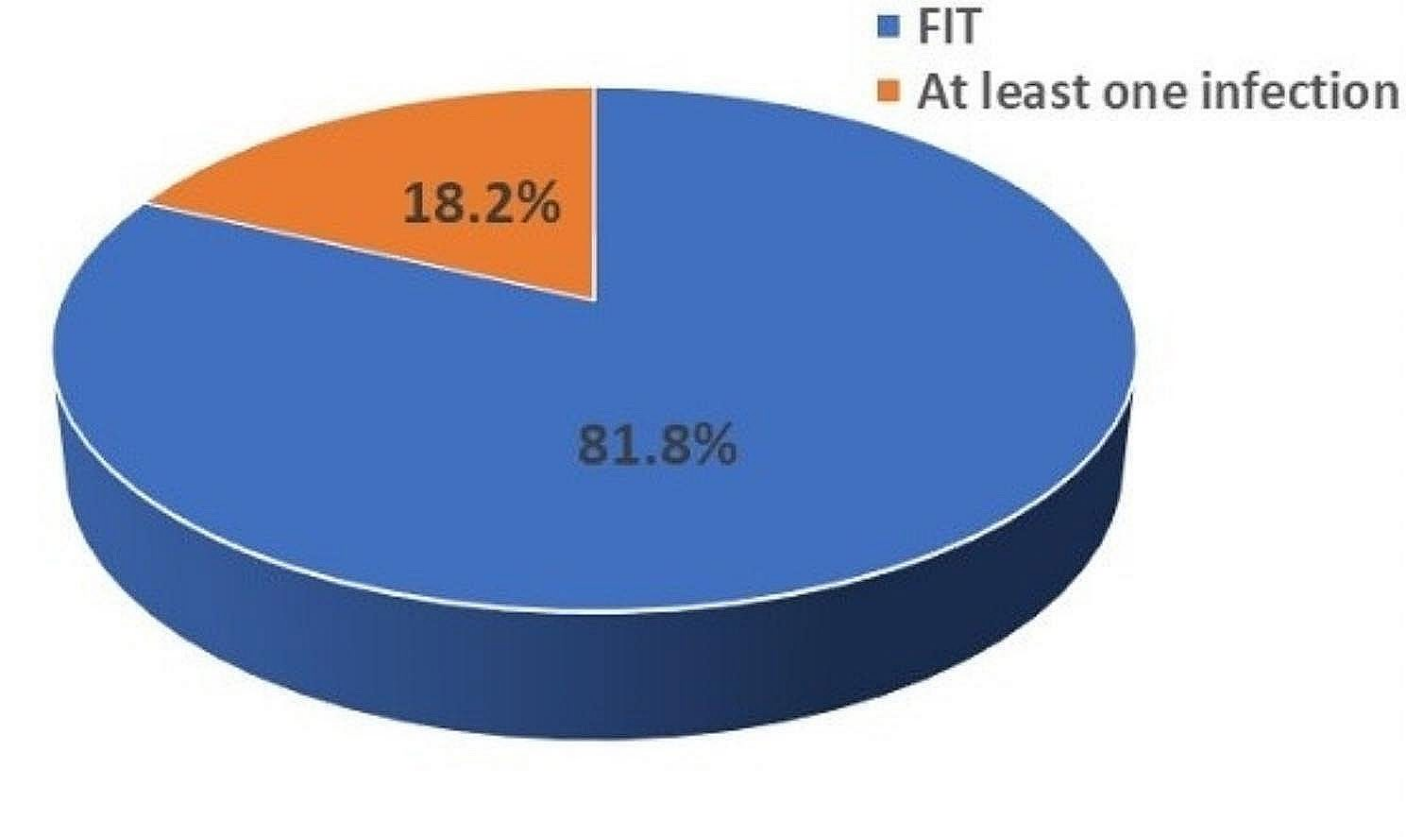
Prevalence of TTIs in the study population

The overall seroprevalence of TTIs in the study population was 17.6%(29/165) using ELISA testing (Fig. 2). Unfit donors with at least one infection was 18.18% (30/165) (amongst which 29 single in infections and 1 co-infection); while 81.81% (135/165) qualified as Fit donors who tested negative for both RDTs and ELISA screening.

The seroprevalence of TTIs were 9.7%and 12.1% for HBSAg, 6.1% and 4.8% for Treponema pallidum, 6.7% and 1.2% for HCV antibodies, 2.4% and 0.6% for HIV antibodies; respectively with RDTs and ELISA (Table 3). The most prevalent TTI using both techniques was HBV.

**Table 3:** Comparing the immunological markers of TTIs between RDT vs. ELISA Techniques

Immunological markers	Results	RDTs N (%)	ELISA N (%)	*p*-value	ODR
**HBsAg**	Negative	149(90.3)	145 (87.9)	< 2,2^e − 16^	-
	Positive	16(9.70)	20 (12.1)		
	Total	165(100)	165(100)		
**HIV antibodies**	Negative	161 (97.6)	164 (99.4)	1	0
	Positive	4 (2.42)	1(0.606)		
	Total	165(100)	165(100)		
***Treponema pallidum*** **antibodies**	Negative	155 (93.9)	157 (95,2)	1.359^− 05^	46.41
	Positive	10(3.63)	8 (4.85)		
	Total	165(100)	165(100)		
**HCV antibodies**	Negative	154 (93.3)	163 (98.8)	1	0
	Positive	11(6,21)	2 (1.21)		
	Total	165 (100)	165 (100)		

## Discussion

Many countries in Sub-Saharan Africa including Cameroon are faced with the challenge of blood safety and availability due to the relatively high prevalence of blood-borne viruses and other infectious diseases prevalent in the region. Therefore, continuous monitoring of the TTIs among the donor population using confirmatory tests is an important index for measuring the effectiveness of existing interventions for better and appropriate control measures.

The preponderance of donors in this study were males and more than 50% were within the age bracket of 18–33 years. A similar pattern has been reported in other regions within Cameroon and indeed other regions in SSA. A six-year review of blood donors at Bamenda regional hospital-based blood bank in Cameroon reported a male donor population of over 85% with 52% being between the ages of 20–29 years [11]. In a similar study, Eboumbou et al. [10] reported a male-to-female ratio of 4:1 among their donor population with the majority of the donors in their third decade of life [10]. In addition, studies in other regions in sub-Saharan Africa have reported similar demographic characteristics among their donor populations [15, 19].

The World Health Assembly resolution WHA63 urges all member states to develop national blood systems based on voluntary unpaid donors. However, in many countries in SSA, family replacement donors (FRD) and paid donors still form a significant pool of their donor population [3]. This finding was corroborated in this study and consistent with studies by Eboumbou et al. [10] and authors in Nigeria, Ghana, and Ethiopia. However, the estimates of FRD in these other studies were much higher than in our study. (89.1%, 96.3% and 88% respectively) [20–22]. Reliance on FRD as a major source of blood supply may affect the timely availability of blood especially in emergency situationswhere family or relatives are not available to donate. Furthermore, blood from paid donors is known to be a major source of TTIs [7, 23].

A major challenge in transfusion practice is donor retention. Lack of repeat donors may impede blood collection and consequently incapacitate the blood transfusion service in maintaining a constant supply of blood. However, in this study, the repeat donors cumulatively were more than the first-time donors. This finding is consistent with the studies by Suemnig et al., [24] and Mauka et al. [25]. In these studies, aimed at determining the factors associated with repeat blood donations by eligible blood donors, the authors reported a donor population of 85.3% vs. 14.3% and 63.9% vs. 36.1% for repeat donors and first-time donors respectively. However, it contrasts with the study by Mohammed et al., [26] who reported more first-time donors than repeat donors (54.9% vs. 45.1%).

Several factors have been suggested as responsible for a donor returning for further donation including altruistic behaviour of the donor, convenient environment and pleasant donation experience, knowledge concerning blood donation and receiving remuneration among others. A combination of these factors may be responsible for the donor behaviour in this study given that there is significant representation of the various types of blood donors reported here.

The overall prevalence of the TTIs in the study was 17.6%. This was slightly higher than the values from similar studies in the littoral region (13.7%) and North West 55555555555 region (10.5%) of Cameroon respectively [10, 11]. It was also slightly higher than the values from studies in Ethiopia (12.4%) [19], Kenya (12%) [27], and Nigeria (13%) [28]. However, it was comparable to studies from Mozambique (18.7%) [29], and Ghana (18.3%) [30], but lower than the values in studies from Wolaita Sodo, Ethiopia (29.5%) [21], Equatorial Guinea (47.4%) [31], and Edea, Cameroon (21.2%) [32].

Several factors may account for these differences in prevalence of these TTIs including differences in the population risk which differ from one country to another and even regions within the same country. Also, the donor recruitment strategies may differ from one country or region to another. Furthermore, the sensitivity and specificity of the various assay techniques used for the detection of the various TTIs may also account for the differences in the prevalence of these infections.

When assessed individually, the prevalence of each of the TTIs using the RDT screening method was relatively higher (HCV-6%, HIV-2.4%, Syphilis-6.1%) than with the ELISA screening technique (HCV-1.2%, HIV-0.6%, Syphilis-4.8%) except for HBV. The ELISA screening technique is widely used in many blood banks as the assay of choice as they are well suited to screening from relatively small to large numbers of samples. Also, they have a higher sensitivity and specificity and are superior to RDT in testing blood donors for TTIs. Furthermore, the ELISA test has a higher positive and negative predictive value and fewer cases of false negative results [8, 33].

Also, from both screening methods (RDT vs. ELISA) HBV infection was the most prevalent TTIs recorded amongst the donors (9.7% and 12.1%). These values are similar to those of some earlier studies in Cameroon. Noubiap et al. [32] reported HBV prevalence of 10.7% among first-time blood donors in Edea, while Sama et al. [34] in a similar study, reported HBV prevalence of 10.1%. Perhaps, the endemicity of this viral infection in sub-Saharan Africa may account for this relatively high prevalence observed in various studies. The overall carrier rate in the general population in this region is 9–20%, and this has been reported to be among the highest globally [35].

The prevalence of HCV in this study was 1.2% and 6.7% using the ELISA and RDT techniques respectively. The trends in this infection among donors vary. While some studies reported a relatively low prevalence others reported otherwise. A study among blood donors in Bamenda Hospital blood service in the Northwest region of Cameroon reported a prevalence of 1.7%, and 1.35% among donors in Laquintinie Hospital in Douala. A similar finding was reported by Macky et al. in Tanzania and Motayo et al. in Southwest Nigeria. On the other hand, Noubiap et al. [31] in Cameroon reported a slightly higher prevalence of 4.2%; and 8.0% infection rate was reported by Bartonjo et al. [12] in Kenya and 3.6% by Okoroiwu et al. [20] in Nigeria. The differences observed in the different studies may be due largely to the assay methodology employed in the detection of the HCV in the donor’s sample as well as the endemic nature of this infection in the locality.

The prevalence of HIV among the donors in this study was 0.6% and 2.4% (ELISA vs. RDT). This value is much less than the population average of 3.7% of adults living with HIV in Cameroon [36]. A similar low prevalence of 1.7% was reported by Eboumbou et al., among blood donors in a hospital blood bank in Douala. However, this contrasts with studies from other regions in Cameroon in which a relatively higher prevalence of 2.2%, 4.1% and 7.5% were reported perhaps due to the use of the RDT method in screening of donors in these studies [11, 31, 38]. The low prevalence rate may be attributed to the robust awareness and sensitization program on HIV/AIDS prevention and control in the region where this study was conducted. These awareness and intervention programs are known to have contributed significantly to the reduction or decline in HIV infection in SSA [37].

Although primarily transmitted through sexual contact, syphilis may also be transmitted via transfusion of blood and blood components donated by asymptomatic donors harbouring the infection and remains a significant threat to safe transfusion practice in many Sub-Saharan countries. The prevalence of 6.1% and 4.8% reported from the use of RDT and TPHA respectively in this study is somewhat worrisome. Earlier studies in Cameroon have reported values similar to the above prevalence [10, 31, 39]. However, the trends from recent studies have reported significantly lesser values: Guekeng et al. [40] 2.31%; Samje et al. [11] reported a prevalence of 2.2%, while a prevalence of 3% was reported among donors in Douala. The relatively high prevalence observed in this study may be due to reduced attention being paid to the disease compared to HIV infection. In addition, paid donors constitute more than 35% of donors in this study. This group of blood donors are known to be high-risk donors with a high potential for harbouring any of the TTIs [23].

Compared to other similar studies, the relatively low sample size in this study constitutes a limitation. This may be attributed to the short duration of the study. Also, responses from the donors could not be verified.

## Conclusion

Transfusion transmissible infections still pose a major health challenge to safe blood transfusion in Mamfe District Hospital given the relatively high prevalence of viral immunological markers detected duly largely to the donor screening method (RDT) adopted. Therefore, we recommend the use of ELISA as the minimum screening method for all donor units in the centre.

## Data Availability

All data generated and analysed in this study are as presented in the result section of the study. Data pertaining to participants personal information are archieved in the hospital blood bank.

## Abbreviations

MDH: Mamfe District Hospital
NBTS: National blood transfusion service
TTIs: Transfusion transmissible infections
SSA: Sub-saharan Africa
VNRD: Voluntary Non-Remunerated Donors
